# Histone H3 lysine 27 acetylation is altered in colon cancer

**DOI:** 10.1186/1559-0275-11-24

**Published:** 2014-06-03

**Authors:** Jakub Karczmarski, Tymon Rubel, Agnieszka Paziewska, Michal Mikula, Mateusz Bujko, Paulina Kober, Michal Dadlez, Jerzy Ostrowski

**Affiliations:** 1 Department of Genetics, Maria Sklodowska-Curie Memorial Cancer Center and Institute of Oncology, Warsaw 02-781, Poland; 2 Institute of Radioelectronics, Warsaw University of Technology, Warsaw 00-665, Poland; 3 Department of Gastroenterology and Hepatology, Medical Center for Postgraduate Education, Warsaw 01-813, Poland; 4 Department of Molecular and Translational Oncology, Maria Sklodowska-Curie Memorial Cancer Center and Institute of Oncology, Warsaw 02-781, Poland; 5 Institute of Biochemistry and Biophysics, Polish Academy of Sciences, Warsaw 02-106, Poland

**Keywords:** Histone, Colorectal cancer, Acetylation, LC-MS

## Abstract

**Background:**

Histone post-translational modifications (PTMs) play an important role in the regulation of the expression of genes, including those involved in cancer development and progression. However, our knowledge of PTM patterns in human tumours is limited.

**Methods:**

MS-based analyses were used to quantify global alterations of histone PTMs in colorectal cancer (CRC) samples. Histones isolated from 12 CRCs and their corresponding normal mucosa by acidic extraction were separated by SDS-PAGE and analysed by liquid chromatography-mass spectrometry.

**Results:**

Among 96 modified peptides, 41 distinct PTM sites were identified, of which 7, 13, 11, and 10 were located within the H2A, H2B, H3, and H4 sequences, respectively, and distributed among the amino-terminal tails and the globular domain of the four histones. Modification intensities were quantified for 33 sites, of which 4 showed significant (*p*-value ≤ 0.05) differences between CRC tissues and healthy mucosa samples. We identified histone H3 lysine 27 acetylation (H3K27Ac) as a modification upregulated in CRC, which had not been shown previously.

**Conclusions:**

The present results indicate the usefulness of a bottom-up proteomic approach for the detection of histone modifications at a global scale. The differential abundance of H3K27Ac mark in CRC, a PTM associated with active enhancers, suggests its role in regulating genes whose expression changes in CRC.

## Background

Colorectal cancer (CRC) is the most common cancer in the Polish population, and the leading cause of cancer-related morbidity and mortality [1]. Most CRCs are sporadic, and only a small proportion is associated with hereditary disorders with high penetration, such as Lynch syndrome, familial adenomatous polyposis and other polyposis syndromes mediated by rare germline mutations in DNA mismatch-repair genes and in the adenomatous polyposis coli (APC) gene [2].

Cancer is a multi-step process involving successive clonal selection events. The growth advantage of dysplastic cells over their normal neighbours leads to progressive cytological and architectural derangement, and individual cancer phenotypes are the result of cell-specific, developmental stage-specific, and metabolism-related changes in gene expression that occur selectively at specific times and are modified by epigenetic interactions [3]. Epigenetic changes such as DNA and histone modifications, chromatin remodelling and regulation by noncoding RNAs can result in massive deregulation of gene expression during the course of cancer development [4]. The global effects of altered epigenetic patterns in gene regulatory sequences have been determined by the ENCODE project [5].

Histone post-translational modifications (PTMs) include lysine acetylation, arginine and lysine methylation, phosphorylation, proline isomerization, ubiquitination (Ub), ADP ribosylation, arginine citrullination, SUMOylation, carbonylation and biotinylation [6]. The most common PTMs are acetylation and methylation [7]. Within the five main histone proteins, PTMs can occur at multiple positions, although they are most frequent at histone N-terminal tails [8]. Despite the key role of epigenetic alterations in cancer development, little is known about the patterns of histone PTM alterations in human tumours [9].

Proteomic methods are largely based on the use of mass spectrometry (MS), a highly specific, effective, and universal technique that does not require complicated multi-step sample preparation. One of the most prominent features of MS is its sensitivity, which enables the detection of attomolar sample concentrations with an error of 0.01% of the total sample mass. Proteomics analyses the composition, amounts, isoforms, and post-translational modifications of cellular proteins [10].

In the present study, we used MS-based analysis to quantify global alterations of histone PTMs in matched normal and colon cancer samples. Our results showed that histone H3 lysine 27 acetylation (H3K27Ac) is associated with colon cancer.

## Results

**Figure 1:**
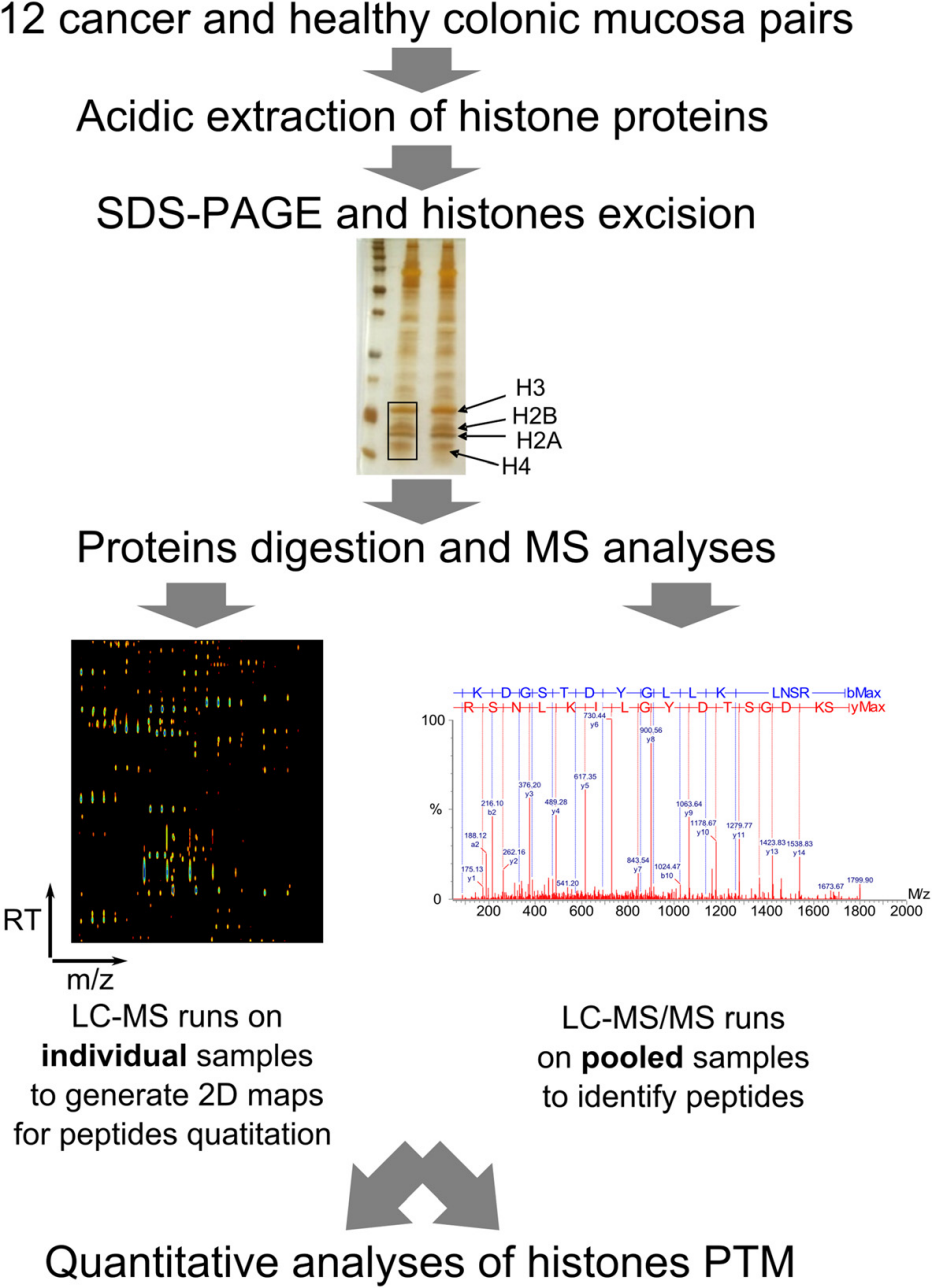
**Diagram of workflow to determine altered histones PTMs in colon carcinoma tissues.** Histones were isolated from whole tissue sections by acidic extraction using Shechter et al. protocol [36] followed by separation using SDS-PAGE and silver staining. The gel part containing histone core proteins were excised next proteins were digested with trypsin and subjected to MS analyses. MS/MS runs of pooled samples were performed to identify in deep the peptides that compose the collection of samples. The custom peptide database was further overlaid on individual 2D maps acquired in LC-MS runs. Maps were then used as the basis to quantify and point modified peptides with differential abundance between CRC and normal mucosa samples.

Histones were isolated from 12 CRC tissues and corresponding normal mucosa, and equal protein amounts were separated by SDS-PAGE and subjected to qualitative LC-MS/MS and quantitative label-free LC-MS analyses (Figure 1).

### Qualitative histone protein analyses

For protein identification 10 pooled samples were analysed by LC-MS/MS for protein identification, resulting in the acquisition of 386120 fragmentation spectra. A search against the SwissProt database using the Mascot engine confidently identified a set of 2,647 peptides with an estimated false discovery rate of 0.01 (Additional file 1: Table S1). In total, 522 proteins were identified, of which 357 were represented by at least two peptides (Additional file 1: Table S2). Among the detected peptides, 285 originated from core histone proteins, including H4, H3.1, H3.2, H3.3 and numerous variants of H2A and H2B. However, the unambiguous identification of the members of the two latter families was difficult. High sequence homology between these two families leads to the detection of multiple shared peptides, which can be attributed to more than a single protein. As a result, it is not always possible to identify the particular proteins present in the samples. In the present study, the indistinguishable histone H2A and H2B subtypes were grouped into six and eight distinct clusters represented by variants accounting for all observed peptides. The final results of core histone protein identification are summarized in Table 1, along with the number of detected peptides and sequence coverage. A more detailed description of their peptide-protein dependencies is also available in Additional file 1: Table S3.

**Table 1:** LC-MS/MS histone protein identification in gel slices

**SwissProt ACC**	**Name**	**Coverage [%]**	**Peptides**	**Modified peptides**	**Modification sites**
**P0C0S8**	**Histone H2A type 1**	**65.89**	**42**	**10**	**5**
**Q96KK5**	**Histone H2A type 1-H**	**66.93**	**42**	**10**	**5**
Q96QV6	Histone H2A type 1-A	66.93	38	6	4
Q93077	Histone H2A type 1-C	65.89	48	10	5
Q6FI13	Histone H2A type 2-A	67.44	43	8	4
Q8IUE6	Histone H2A type 2-B	50.39	13	2	1
**Q71UI9**	**Histone H2A.V**	**50.39**	**13**	**2**	**1**
**P0C0S5**	**Histone H2A.Z**	**50.39**	**13**	**2**	**1**
P16104	Histone H2A.x	65.49	43	9	5
P62807	Histone H2B type 1-C/E/F/G/I	86.40	51	19	13
P58876	Histone H2B type 1-D	86.40	50	18	13
Q93079	Histone H2B type 1-H	86.40	51	19	13
P06899	Histone H2B type 1-J	86.40	50	19	13
Q99880	Histone H2B type 1-L	80.80	49	18	13
P23527	Histone H2B type 1-O	86.40	50	19	13
Q16778	Histone H2B type 2-E	86.40	50	19	13
**P57053**	**Histone H2B type F-S**	**86.40**	**51**	**19**	**13**
**O60814**	**Histone H2B type 1-K**	**86.40**	**51**	**19**	**13**
P68431	Histone H3.1	80.00	61	28	10
Q71DI3	Histone H3.2	80.00	59	30	10
P84243	Histone H3.3	80.00	55	27	11
P62805	Histone H4	80.39	42	12	11

Proteins matching the same sets of peptides are indicated by bolded text. Non-histone proteins are not listed.

**Figure 2:**
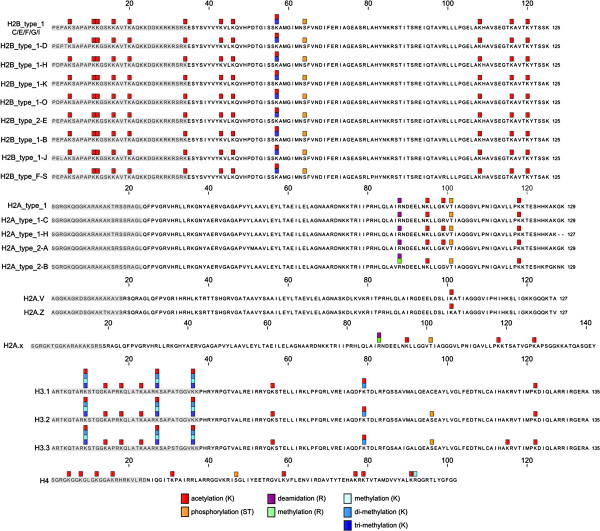
**Amino acid sequences of core histone and their forms, indicating sites of post-translational modification.** The grey block marks the N-terminal histone tail. The alignment of protein sequences and visualization of the detected modification sites were performed using CLC Sequence Viewer (CLC Bio).

The identification of 96 modified peptides allowed for the characterisation of 41 distinct post-translational modification sites, of which 7, 13, 11, and 10 were located within the sequences of H2A, H2B, H3, and H4, respectively (Additional file 1: Table S4 and Figure 2). Multiple modification variants were detected on 6 sites (14.6%), with a maximum of four different modifications per site. As shown in Figure 2, the sites were distributed in the amino-terminal tails and the globular domain forming the nucleosomal core of each of the four histones. Despite the sequence divergence, the observed modification patterns were generally preserved within the H2A, H2B and H3 families, with the exception of H2A.V and H2A.Z variants.

Acetylation, which was observed in 36 lysine residues, was the most prominent of all the studied modifications. In 31 instances, it was the only modification detected on a given site, whereas the remaining 5 sites showed alternative modification variants, including mono-, di- and tri-methylation (three, four, and four sites, respectively). Modifications on arginine residues were significantly less frequent and only a single site of methylation and deamidation was detected in the sequence of the histone H2A. Two serine and two threonine phosphorylation sites were also identified.

A crosscheck with the PhosphoSitePlus [11] and Histome [12] databases revealed that although most of the sites had been previously reported, in several cases our survey provided a more comprehensive list of their possible modification. For example, lysine K43 of histone H2B, which is a known ubiquitination site, was shown to have an acetylated variant. A detailed summary of site-modification combinations not covered by the two databases is presented in Additional file 1: Table S4.

### Quantitative analysis

Peptides identified by LC-MS/MS analyses were quantified in individual samples (12 CRC tissue-healthy mucosa pairs) using a label-free approach and modification intensities were calculated for each of the detected sites (see Methods). We obtained 45 reliable quantitative estimates of modification intensities on 33 sites. Using a threshold of *p* ≤ 0.05, 4 sites exhibiting significant differences in modification intensity between the two sample groups were detected (Table 2), of which three were upregulated and one downregulated in cancerous samples. Among them we identified histone H3 lysine 27 acetylation (H3K27Ac) as a modification upregulated in CRC; an example of fragmentation spectrum for H3K27Ac is presented in Additional file 2: Figure S1.

**Table 2:** **List of histone modifications showing differences in abundance (*p*-value ≤ 0.05) between cancerous (CRC) and normal (NC) colonic mucosa**

**Histone**	**Site**	**Modification**	***p*-value**	**FC CRC/NC**
H3	K27	Trimethyl (K)	0.004032	1.67
H3	K27	Acetyl (K)	0.012378	1.54
H2B	S64	Phospho (ST)	0.016537	1.24
H3	K79	Acetyl (K)	0.044106	0.83

FC, fold change.

**Figure 3:**
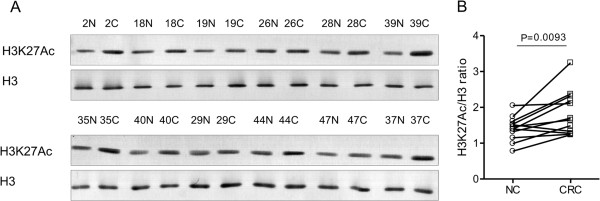
**H3K27Ac is upregulated in colon carcinoma tissues. (A)** Results of H3 and H3K27Ac immunostaining in 12 pairs of CRC tissues and normal mucosa and graphical depiction of densitometric assessment of differences **(B)**. Histones were isolated from whole tissue sections by acidic extraction and equal amounts of protein were separated by SDS-PAGE, electrotransferred to PVDF membranes and immunostained with H3 (ab1791) or H3K27Ac (ab4729) antibody. Densitometric measurements were performed using OptiQuant image analysis software. H3K27Ac level was normalised to the signal from total H3. C, CRC- colon cancer; N, NC- normal colonic mucosa.

Availability of antibodies allowed further evaluation of the two selected PTMs of histone H3, namely H3K27Ac and K27 trimethylation (me3). Western blotting-based analysis of histones isolated from 12 CRC tissue samples and paired healthy mucosa samples, followed by densitometric analyses, confirmed increased K27 acetylation at H3 (fold change (FC) = 1.31, *p-*value = 0.0093) (Figure 3), whereas no significant differences were observed for the H3K27me3 marker (not shown).

**Figure 4:**
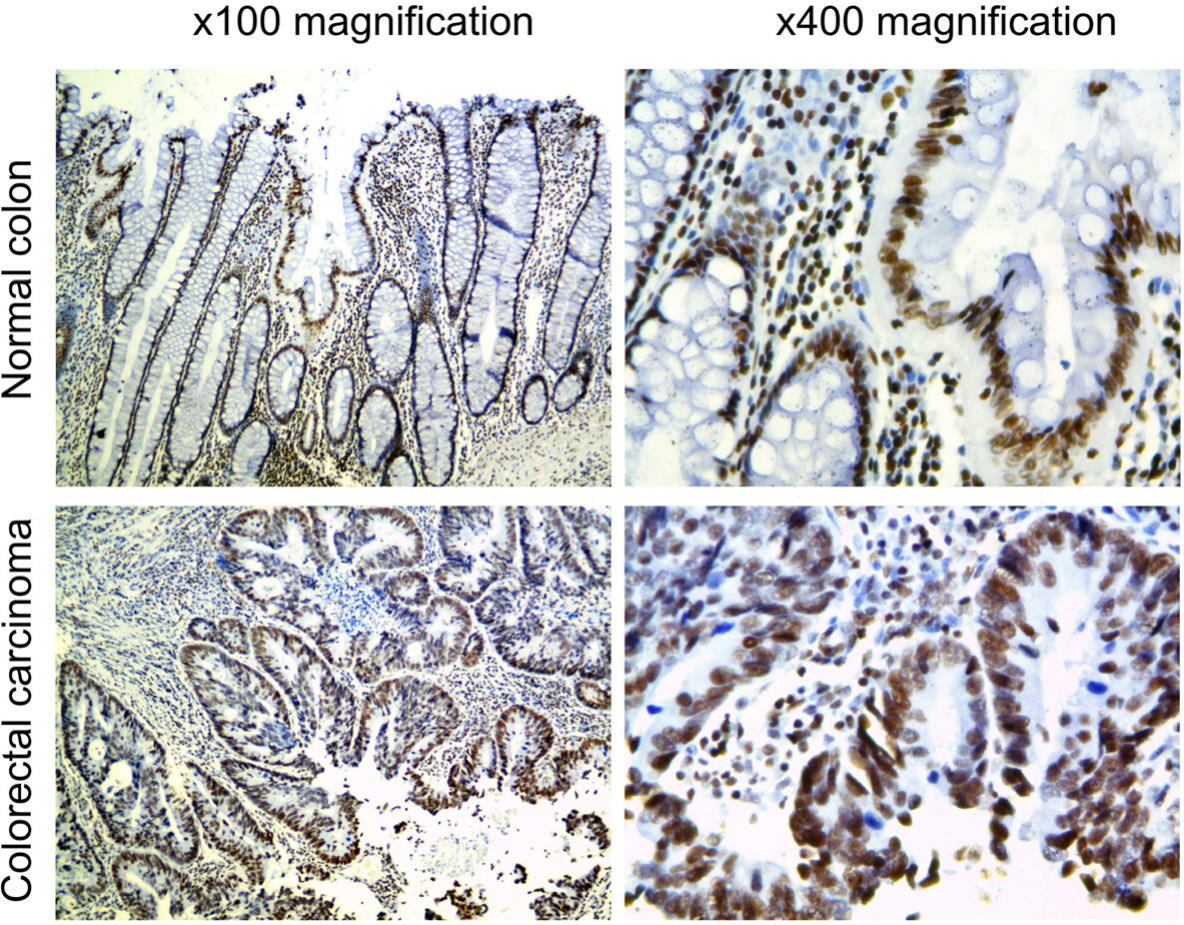
An example of an immunohistochemical analysis of representative matched normal colonic and CRC sections with use of antibody against H3K27Ac.

MS and western blot results of H3K27Ac alteration were also confirmed by the immunohistochemical staining of 10 pairs of normal and CRC formalin-fixed paraffin-embedded tissue samples; five tissue pairs were common with MS and western blot analyses (Additional file 1: Table S5). While both normal and CRC tissue revealed pronounced positive nuclear immunoreactivity for H3K27Ac (Figure 4 and Additional file 2: Figure S2), the percentage of positively stained nuclei (labelling index), calculated with the use of automated image analysis software in the representative pictures of matched normal/CRC sample sections, revealed higher ratio of immunopositive cells compared to normal counterpart (69.01% vs. 52.66% respectively, p = 0.0052) - Table 3.

**Table 3:** H3K27Ac labelling index in matched CRC and corresponding normal tissue sections

**Sample ID**^**#**^	**CRC tissue**	**Normal colonic section**
11028	60.9	67.1
14585	59.5	55.6
14600	55.2	54.9
14616	75.1	46.5
14882	75.7	39.2
14935	62.2	43
15244	78.5	49.3
17022	79.9	53.9
10991	89	62.4
15732	54.1	54.7

#details on the samples can be found in Additional file 1: Table S5.

To determine whether increase in H3K27Ac mark is associated with proliferating cells, we measured its abundance in four resting and dividing CRC cell lines, namely HCT-116, Colo205, HT29 and Caco2, using the western blot. While there were no differences in H3K27Ac levels between quiescent and proliferating cells, variable levels of that mark were found between the cell lines (Additional file 2: Figure S3).

Next, we wished to determine the expression of enzymes controlling H3K27Ac mark. To this end, using quantitative (q) RT-PCR, we measured the mRNA levels of enzymes controlling H3K27Ac mark abundance, namely CBP/p300 acetyltransferases [13,14] and HDAC1 deacetylase [15], on 26 CRC and 24 healthy mucosa samples collected in our previous study [16]. These measurements revealed significant downregulation of CBP, p300 and HDAC1 transcripts in CRC (Additional file 2: Figure S4) with a FC of 0.37, 0.34 and 0.7, respectively.

## Discussion

Histone PTMs can affect DNA-histone interactions or inter-nucleosomal contacts, as well as the recruitment of non-histone proteins to chromatin *via* bromo, chromo and PHD domains [17,18]. Thus, PTMs are responsible for the regulation of chromatin structure and function, constituting the epigenetic code. Although challenging, traditional MS-based bottom-up analyses allow the identification and characterisation of PTMs without prior knowledge of the modification site or type (reviewed in [6]).

In the present study, we used a bottom-up proteomic approach to investigate alterations in histone modifications in colon cancer samples and their normal counterparts. We identified 96 modified histone derived peptides, of which 45 site-modification combinations were further quantified, revealing 4 sites with differential abundance between cancerous and normal mucosa. For further validation, we chose histone H3 lysine 27 acetylation because this modification has not been previously shown to be altered in CRC [19].Protein immunostaining on western blots confirmed the increased K27 acetylation at H3 (Figure 3) and immunohistochemical staining of paired CRC and corresponding normal tissue sections revealed nuclear localisation of acetylated H3 protein (Figure 4). Since different morphology of cells of epithelial type is observed in two types of tissue, the percentage of immunoractive cells (labelling index) was calculated in the representative pictures of each sample and used for the comparison of the two types of colorectal tissue. We found significantly higher H3K27Ac index in CRC samples compared to normal tissue which correspond the results obtained with both of MS and western blot.

Acetylation of lysine residues is a major histone modification involved in the regulation of chromatin structure and transcription. It neutralises the positive charge on the lysine side chain, relaxing the chromatin structure, and it generates docking sites for bromodomain-containing proteins [20]. The balance between the enzymatic activities of histone lysine acetyltransferases and deacetylases regulates the level of histone acetylation. Furthermore, the global level of histone acetylation depends on intracellular acetyl-CoA pools [21].

Acetyltransferases consist of three families, GNAT, MYST, and CBP/p300, which generally act promiscuously on more than one lysine; however, some specificity has been observed for these enzymes [20]. Aberrant acetylation of histones has been linked to CRC pathogenesis (reviewed in [19]). Studies suggest that histone acetylation is reduced in CRC and in other tumours [22]; however, examination of specific sites shows that acetylation can be either up- or downregulated. For example, Fraga et al. used EC-LC-ES/MS and western blot analysis and showed a loss of monoacetylation at H4K16 in CRC cell lines [23]. Global hypoacetylation of H4K12 and H3K18 has been observed in undifferentiated colorectal adenocarcinomas, whereas their acetylation was increased in well-differentiated tumours [24]. Contrary to these findings, the H3K9 hypoacetylation status was positively correlated with tumour histological type and low H3K9Ac was observed in well-differentiated tumours [25]. To the best of our knowledge, aberrant H3K27Ac levels in CRC have not been reported to date. In other solid tumours, namely lung adenocarcinomas and squamous cell carcinomas, the increase in H3K27 acetylation is more pronounced in the tumour compartment than in the corresponding stroma [26].

H3K27Ac was first discovered in yeast [27] and is present in animals and plants [28]. Recent advancements in DNA sequencing technology have enabled the analysis of histone modification distribution patterns across the genome. These studies have shown that among histone acetylation marks [29], H3K27Ac is frequently associated with active enhancer regulatory elements [30], and genes associated with these enhancers are expressed at higher levels than those lacking the H3K27Ac mark [31]. The H3K27Ac mark is established by CBP/p300 acetyltransferase [13,14] and is likely erased by RBP3/HDAC1 [15]. Contrary to the report by Ishihama et al. who found CBP/p300 and HDAC1 mRNAs [32] upregulated in CRC, our qPCR measurements showed decreased levels of these transcripts (Additional file 2: Figure S4). The discrepancy could be due to the differences in the methodologies of transcripts measurements; while Ishihama et al. used both semi-quantitative endpoind RT-PCR and in situ hybridisation [32], in this study we used qPCR which is regarded as a gold standard for the quantification of nucleic acids [33]. Although other reports highlighted higher abundances of CBP/p300 and HDAC1 proteins in CRC tissue [34,35], it must be stressed that the alterations in the expression of these epigenetic enzymes are not entirely responsible for specific histone modification levels, because other variables such as substrate availability and enzyme activity may contribute to the final modification.

## Conclusions

In conclusion, for the first time, we show that H3K27Ac mark is increased in CRC. Further experiments, which are beyond the scope of this study, such as chromatin immunoprecipitation followed by deep sequencing (ChIP-Seq) in colon tissues to examine the distribution of the H3K27Ac mark, may identify enhancer regulatory sites that differ in mark levels between normal and cancerous samples. Such datasets combined with increased knowledge on genome regulatory elements and long genomic interactions deposited in the ENCODE database [5] could potentially lead to the identification of genes whose expression changes are associated with altered H3K27Ac status.

## Methods

### Tissue samples

Patients were selected as described previously [4]. The study protocol was approved by the Cancer Center Bioethics Committee, and all patients signed informed consent forms before inclusion. Twelve sporadic CRC samples and paired full-thickness normal colon fragments were obtained by surgical resection through laparotomy, snap frozen in liquid nitrogen within 10–30 min of harvesting and stored at 72°C until use. To select samples with a high content of normal and dysplastic mucosa, several series of cryostat sections were prepared from different parts of each specimen using a Microm HM 505E (Zeiss, Germany). Histological evaluation of the upper and lower sections from each cryosection collection revealed a 70% median-relative content of normal mucosa (range 40 − 90%) and 50% (20 − 90%) dysplastic mucosa in the specimens. Clinical characteristics and histopathology of the tissue samples used in a study is presented in Additional file 1: Table S5.

### Colon adenocarcinoma cell lines

HCT-116, Colo205, HT-29 and Caco2 cell lines were purchased from ATCC and maintained under standard conditions in culture medium (HCT-116 and HT-29 – McCoy’s 5A Medium; Colo205 – RPMI-1650 medium; Caco2 – DMEM medium) supplemented with 10% FBS (Fetal Bovine Serum). Cells were made quiescent by lowering FBS concentration to 0.5% FBS for 24 h prior to harvest.

### Histones extraction

Histones were isolated from whole tissue sections by H_2_SO_4_ extraction [36] and equal amounts of sample protein (20 μg) were separated by standard 15% SDS-PAGE and silver stained. Gel fragments containing H2A, H2B, H3, and H4 bands were cut out separately for each electrophoresis line; proteins in the gel were reduced, alkylated, and trypsin-digested using standard protocols, and the resulting peptides were extracted using 0.1% TFA/2% acetonitrile (ACN).

### Western blot and densitometric data analyses

For western blotting, 5 μg of each protein sample was resolved by SDS-PAGE and electrotransferred to a PVDF membrane. Blotted proteins were detected using the following antibodies: H3 (ab1791, Abcam), H3K27Ac (ab4729, Abcam), H3K27me3 (07–449, Millipore). Protein band intensities were assessed by densitometry using OptiQuant image analysis software (Packard). Data were normalised to the amount of total histone H3 and expressed as fold change in CRC tissues versus control samples. The statistical significance of histone modifications between two tissue types, as detected by western blotting, was determined with the nonparametric Wilcoxon signed-rank test, and *p*-values of ≤ 0.05 were considered significant.

### Quantitative RT-PCR

Measurements of mRNA abundances were performed on the 26 CRC and 24 healthy mucosa samples and with a q-RT-PCR protocol described previously [16]. The sequences of primers used were as follow: HDAC1, forward 5′- CAATGAAGCCTCACCGAATC-3′, reverse 5′- CTCCTCAGCATTGGCTTTGT-3′; CBP, forward 5′- GTTTCCCCGCAAATGACTG-3′, reverse 5′- CTGCCCTCCAGCTTGACTAA-3′; p300, forward 5′- CATGAATCCAGGGCCTAACA-3′, reverse 5′- CGGATCATACTTGGGTCAGG-3′.

### Immunohistochemistry

Staining was performed in 4 μm formalin-fixed, paraffin-embedded tissue sections of CRCs and matched normal mucosa from 10 patients (Additional file 1: Table S5) with the use of Envision Detection System (DAKO). Sections were deparaffinized with xylene and rehydrated in a series of decreasing concentration of ethanol solutions. Heat-induced epitope retrieval was carried out in Target Retrieval Solution (pH 6) (DAKO) in a 96°C water bath, for 20 minutes. After cooling retrieval solutions for 25 minutes at room temperature, the slides were treated for 5 minutes with Blocker of Endogenous Peroxidase (DAKO). Slides were incubated with anti-H3K27Ac (ab4729, Abcam) (diluted 1:500) for 30 minutes at room temperature and subsequently labeled with the Envision Detection System (DAKO). Color reaction product was developed with 3,3′-diaminobenzidine, tetrahydrochloride (DAB) (DAKO) as a substrate, and nuclear contrast was achieved with hematoxylin counterstaining. Representative pictures of each sample were taken at magnification x400 and used for the automatic calculation of the percentage of positively stained nuclear area (labeling index). For this purpose ImmunoRatio i.e. softwere for automated image analysis [37] was used and the results for normal samples and CRC sections were compared.

### LC-MS settings

LC-MS analysis of histone peptides was performed used a LTQ-Orbitrap Velos mass spectrometer (Thermo Scientific) coupled with a nanoAcquity (Waters Corporation) LC system. Spectrometer parameters were as follows: polarity mode, positive; capillary voltage, 1.5 kV. A sample was first applied to the nanoACQUITY UPLC Trapping Column (Waters) using water containing 0.1% formic acid as the mobile phase. Next, the peptide mixture was transferred to the nanoACQUITY UPLC BEH C18 Column (Waters, 75 μm inner diameter; 250 mm long) and an ACN gradient (5–40% over 100 min) was applied in the presence of 0.1% formic acid with a flow rate of 250 nl/min and eluted directly to the ion source of the mass spectrometer. Each LC run was preceded by a blank run to avoid sample carry-over between the analyses.

Qualitative LC-MS/MS analyses were performed on pooled samples in data-dependent acquisition mode. Up to 5 MS/MS processes were allowed for each MS scan, and high-energy collision dissociation (HCD) was used for peptide fragmentation. Quantitative analyses of individual samples were performed by using separate survey scan LC-MS runs with a m/z measurement range of 300–2,000 and the same ACN gradient settings as those used for the LC-MS/MS runs. The data-dependent MS-to-MS/MS switch was disabled, and the spectrometer resolution was set to 15,000.

### Qualitative MS data processing and database search

The acquired MS/MS raw data files were preprocessed with Mascot Distiller (version 2.2.1, Matrix Science), and the resulting peak lists were searched against the Homo sapiens entries of the SwissProt database (version 05.10.2012, 20306 sequences) using Mascot (version 2.2.03, Matrix Science). The search parameters were as follows: enzyme specificity: semitrypsin; maximum number of missed cleavages: 2; protein mass: unrestricted; parent ions mass error tolerance: 5 ppm; fragment ions mass error tolerance: 0.02 Da; fixed modifications: Carbamidomethylation (C); and variable modifications: Acetyl (K) (42.010565 Da), Methyl (K) (14.015650 Da), Dimethyl (K) (28.031300 Da), Trimethyl (K) (42.046950 Da), Methyl (R) (14.015650 Da), Dimethyl (R) (28.031300 Da), Deamidated (R) (0.984016 Da), Phospho (ST) (79.966331 Da) and Oxidation (M) (15.994915 Da).

The statistical significance of peptide identifications was determined using a target/decoy database search approach and a previously described procedure that provided *q*-value estimates for each peptide spectrum match (PSMs) in the data set [38,39]. Only PSMs with *q*-values ≤ 0.01 were regarded as confidently identified.

Additional acceptance criteria were used for assessing confidence of modified peptides. In the first step, the exact position of the modifications in the sequence was established by an adopted version of the phosphoRS algorithm [40]. Next, the MS/MS spectra were inspected manually for accurate fragment ions assignment. Finally, selected types of sites were rejected as potential experimental artifacts. Those included: lysine methylations on the C-terminus of the sequence or detected in peptides with acidic residues (possible artifacts of methyl esterification of the carboxylic group) and peptides with deamidation on the C-terminal arginine (tryptic cleavage after a deamidated residue have been recently shown as a highly unlikely event [41]).

Proteins represented by less than two peptides, or identified by a subset of peptides from another protein, were excluded from further analysis. Proteins matching the same set of peptides were grouped together into clusters. All the steps involved in Mascot results processing were performed using MScan, a proprietary Java application available at http://proteom.ibb.waw.pl/mscan. Multiple alignment of protein sequences and visualization of the detected post-translational modification sites were performed using CLC Sequence Viewer (CLC Bio).

### Quantitative MS data processing

Peptides identified in all LC-MS/MS runs were merged into a common list, which was next overlaid onto 2-D maps generated from the LC-MS profile data of individual samples. The feature extraction procedure was described in detail in a previous study [42]. Briefly, the list of identified peptides was used to tag the corresponding peptide-related ion spectra based on m/z differences, deviations from the predicted elution times, and the match between the theoretical and observed isotopic envelopes. The maximum deviation accepted in m/z and the retention time were established separately for each of the processed LC-MS spectra to account for possible variations in mass measurement accuracy and chromatographic separation between runs. First, an initial search with wide tolerance and restrictive parameters of isotopic envelope fits was performed. Next, nonlinear mass and time calibration functions were calculated using LOESS regression, and the search was repeated with narrowed tolerances and relaxed fit requirements. Finally, relative abundances of peptide ions were determined as the heights of 2-D fits to the most prominent peaks of the tagged isotopic envelopes. For normalisation purposes, the calculated abundance of each peptide was divided by the median abundance of all the peptides detected in the sample.

Given the normalised peptide abundances, quantitative values (further referred to as “modification intensities”) were calculated for distinct post-translational modification types observed on each of the previously identified sites. These values were computed using a procedure that involved rescaling of the abundances of single-modified peptides covering the site of interest to a common level, followed by computing their median value.

### Statistical analysis of quantitative MS measurements

A non-parametric resampling-based test with paired *t* statistics was used to evaluate the differences in site-modification intensities between the two groups of samples. Modification sites with *p*-values ≤ 0.05 were considered as significantly changed.

## Competing interests

The authors declare that they have no competing interests.

## Authors’ contributions

JO designed and supervised the study, wrote the manuscript; JK and AP carried out the experiments and MS runs; TR analyzed MS data and wrote the manuscript; MM interpreted the data and drafted the manuscript; MB and PK performed and analyzed immunohistochemistry data; MD supervised MS runs. All authors read and approved the final manuscript.

## Supplementary Material

Additional file 1: Table S1 The list of peptides identified in MS/MS analyses with estimated FDR = 0.01. **Table S2.** The list of identified proteins together with their MS/MS-related details. SCORE - MascotScore; PEPT# - number of peptides assigned to protein. Proteins matching to the same sets of peptides were grouped into unique clusters and presented as single rows of the table. **Table S3.** List of identified core histone-derived peptides. **Table S4.** List of identified postranslational modification sites core histone proteins. Site-modification combinations not present in PhopshoSitePlus and/or Histome databases are marked in red. **Table S5.** Clinical characteristics and histopathology of the tissue samples used in a study. Click here for file

Additional file 2: Figure S1 An example MS/MS spectrum of the peptide K(Ac)SAPATGGVK derived from the H3 histone proteins family. The amino acid sequence of the peptide includes the lizyne K27 residue. The plot was generated using the ExpertSystemGui application available at (http://www.biochem.mpg.de/mann/tools/). **Figure S2.** Immunohistochemical staining of 10 matched normal and CRC tissue sections with use of antobody against H3K27Ac. Magnifications 100X and 400X. **Figure S3.** H3K27Ac mark level in quiescent and proliferating CRC cell lines. Cells cultured for 24 h with 10% or 0.5% FBS were harvested, histones isolated by acidic extraction and then 5 μg of protein was resolved by SDS-PAGE and electrotransferred to PVDF membrane. Blotted proteins were assessed by Western blot analysis using the antibodies to H3 (ab1791) or H3K27Ac (ab4729). Densitometric measurements were performed using OptiQuant image analysis software. H3K27Ac level was normalised to the signal from total H3 and presented of the chart. **Figure S4.** Analysis of mRNA expression levels of CBP, p300 and HDAC1 in individual tissue samples of 26 adenocarcinomas (CRC) and 24 healthy mucosa’s (NC). One microgram of total RNA was reverse-transcribed to generate cDNA and then qPCR was performed using SYBR Green I chemistry. Green horizontal bars indicate means and red whiskers indicate standard deviation. Differences were analyzed using the Mann–Whitney test. Click here for file

## References

[B1] Gaj P Maryan N Hennig EE Ledwon JK Paziewska A Majewska A Karczmarski J Nesteruk M Wolski J Antoniewicz AA Przytulski K Rutkowski A Teumer A Homuth G Starzyńska T Regula J Ostrowski J Pooled sample-based GWAS: a cost-effective alternative for identifying colorectal and prostate cancer risk variants in the polish population PLoS ONE 2012 11 e35307 22532847 10.1371/journal.pone.0035307 PMC3331859

[B2] Peters U Hutter CM Hsu L Schumacher FR Conti DV Carlson CS Edlund CK Haile RW Gallinger S Zanke BW Lemire M Rangrej J Vijayaraghavan R Chan AT Hazra A Hunter DJ Ma J Fuchs CS Giovannucci EL Kraft P Liu Y Chen L Jiao S Makar KW Taverna D Gruber SB Rennert G Moreno V Ulrich CM Woods MO Meta-analysis of new genome-wide association studies of colorectal cancer risk Hum Genet 2012 11 217 234 21761138 10.1007/s00439-011-1055-0 PMC3257356

[B3] Weichenhan D Plass C The evolving epigenome Hum Mol Genet 2013 11 R1 6 23900077 10.1093/hmg/ddt348

[B4] Skrzypczak M Goryca K Rubel T Paziewska A Mikula M Jarosz D Pachlewski J Oledzki J Ostrowski J Ostrowski J Modeling oncogenic signaling in colon tumors by multidirectional analyses of microarray data directed for maximization of analytical reliability PLoS ONE 2010 11 e13091 20957034 10.1371/journal.pone.0013091 PMC2948500

[B5] Bernstein BE Birney E Dunham I Green ED Gunter C Snyder M ENCODE Project Consortium An integrated encyclopedia of DNA elements in the human genome Nature 2012 11 57 74 22955616 10.1038/nature11247 PMC3439153

[B6] Arnaudo AM Garcia BA Proteomic characterization of novel histone post-translational modifications Epigenetics Chromatin 2013 11 24 23916056 10.1186/1756-8935-6-24 PMC3737111

[B7] Tan M Luo H Lee S Jin F Yang JS Montellier E Buchou T Cheng Z Rousseaux S Rajagopal N Lu Z Ye Z Zhu Q Wysocka J Ye Y Khochbin S Ren B Zhao Y Identification of 67 histone marks and histone lysine crotonylation as a new type of histone modification Cell 2011 11 1016 1028 21925322 10.1016/j.cell.2011.08.008 PMC3176443

[B8] Tropberger P Schneider R Going global: novel histone modifications in the globular domain of H3 Epigenetics 2010 11 112 117 20160509 10.4161/epi.5.2.11075

[B9] Migheli F Migliore L Epigenetics of colorectal cancer Clin Genet 2012 11 312 318 22263639 10.1111/j.1399-0004.2011.01829.x

[B10] Ostrowski J Wyrwicz LS Integrating genomics, proteomics and bioinformatics in translational studies of molecular medicine Expert Rev Mol Diagn 2009 11 623 630 19732006 10.1586/erm.09.41

[B11] Hornbeck PV Kornhauser JM Tkachev S Zhang B Skrzypek E Murray B Latham V Sullivan M PhosphoSitePlus: a comprehensive resource for investigating the structure and function of experimentally determined post-translational modifications in man and mouse Nucleic Acids Res 2012 11 Database issue D261 270 22135298 10.1093/nar/gkr1122 PMC3245126

[B12] Khare SP Habib F Sharma R Gadewal N Gupta S Galande S HIstome–a relational knowledgebase of human histone proteins and histone modifying enzymes Nucleic Acids Res 2012 11 Database issue D337 342 22140112 10.1093/nar/gkr1125 PMC3245077

[B13] Wolf L Harrison W Huang J Xie Q Xiao N Sun J Kong L Lachke SA Kuracha MR Govindarajan V Brindle PK Ashery-Padan R Beebe DC Overbeek PA Cvekl A Histone posttranslational modifications and cell fate determination: lens induction requires the lysine acetyltransferases CBP and p300 Nucleic Acids Res 2013 11 10199 214 24038357 10.1093/nar/gkt824 PMC3905850

[B14] Jin Q Yu L-R Wang L Zhang Z Kasper LH Lee J-E Wang C Brindle PK Dent SYR Ge K Distinct roles of GCN5/PCAF-mediated H3K9ac and CBP/p300-mediated H3K18/27 ac in nuclear receptor transactivation EMBO J 2011 11 249 262 21131905 10.1038/emboj.2010.318 PMC3025463

[B15] Tie F Banerjee R Stratton CA Prasad-Sinha J Stepanik V Zlobin A Diaz MO Scacheri PC Harte PJ CBP-mediated acetylation of histone H3 lysine 27 antagonizes drosophila polycomb silencing Development 2009 11 3131 3141 19700617 10.1242/dev.037127 PMC2730368

[B16] Mikula M Rubel T Karczmarski J Goryca K Dadlez M Ostrowski J Integrating proteomic and transcriptomic high-throughput surveys for search of new biomarkers of colon tumors Funct Integr Genomics 2011 11 215 224 10.1007/s10142-010-0200-5 21061036

[B17] Leroy G Dimaggio PA Chan EY Zee BM Blanco MA Bryant B Flaniken IZ Liu S Kang Y Trojer P Garcia BA A quantitative atlas of histone modification signatures from human cancer cells Epigenetics Chromatin 2013 11 20 23826629 10.1186/1756-8935-6-20 PMC3710262

[B18] Izzo A Schneider R Chatting histone modifications in mammals Brief Funct Genomics 2010 11 429 443 21266346 10.1093/bfgp/elq024 PMC3080777

[B19] Gargalionis AN Piperi C Adamopoulos C Papavassiliou AG Histone modifications as a pathogenic mechanism of colorectal tumorigenesis Int J Biochem Cell Biol 2012 11 1276 1289 22583735 10.1016/j.biocel.2012.05.002

[B20] Kouzarides T Chromatin modifications and their function Cell 2007 11 693 705 17320507 10.1016/j.cell.2007.02.005

[B21] Wellen KE Hatzivassiliou G Sachdeva UM Bui TV Cross JR Thompson CB ATP-citrate lyase links cellular metabolism to histone acetylation Science 2009 11 1076 1080 19461003 10.1126/science.1164097 PMC2746744

[B22] Chen C Zhao M Yin N He B Wang B Yuan Y Yu F Hu J Yin B Lu Q Abnormal histone acetylation and methylation levels in esophageal squamous cell carcinomas Cancer Invest 2011 11 548 556 21843048 10.3109/07357907.2011.597810

[B23] Fraga MF Ballestar E Villar-Garea A Boix-Chornet M Espada J Schotta G Bonaldi T Haydon C Ropero S Petrie K Iyer NG Pérez-Rosado A Calvo E Lopez JA Cano A Calasanz MJ Colomer D Piris MA Ahn N Imhof A Caldas C Jenuwein T Esteller M Loss of acetylation at Lys16 and trimethylation at Lys20 of histone H4 is a common hallmark of human cancer Nat Genet 2005 11 391 400 15765097 10.1038/ng1531

[B24] Ashktorab H Belgrave K Hosseinkhah F Brim H Nouraie M Takkikto M Hewitt S Lee EL Dashwood RH Smoot D Global histone H4 acetylation and HDAC2 expression in colon adenoma and carcinoma Dig Dis Sci 2009 11 2109 2117 19057998 10.1007/s10620-008-0601-7 PMC2737733

[B25] Tamagawa H Oshima T Shiozawa M Morinaga S Nakamura Y Yoshihara M Sakuma Y Kameda Y Akaike M Masuda M Imada T Miyagi Y The global histone modification pattern correlates with overall survival in metachronous liver metastasis of colorectal cancer Oncol Rep 2012 11 637 642 22076537 10.3892/or.2011.1547

[B26] Roche J Nasarre P Gemmill R Baldys A Pontis J Korch C Guilhot J Ait-Si-Ali S Drabkin H Global decrease of histone H3K27 acetylation in ZEB1-induced epithelial to mesenchymal transition in lung cancer cells Cancers 2013 11 334 356 24216980 10.3390/cancers5020334 PMC3730320

[B27] Suka N Suka Y Carmen AA Wu J Grunstein M Highly specific antibodies determine histone acetylation site usage in yeast heterochromatin and euchromatin Mol Cell 2001 11 473 479 11545749 10.1016/s1097-2765(01)00301-x

[B28] Tie F Banerjee R Conrad PA Scacheri PC Harte PJ Histone demethylase UTX and chromatin remodeler BRM bind directly to CBP and modulate acetylation of histone H3 lysine 27 Mol Cell Biol 2012 11 2323 2334 22493065 10.1128/MCB.06392-11 PMC3372260

[B29] Wang Z Zang C Rosenfeld JA Schones DE Barski A Cuddapah S Cui K Roh T-Y Peng W Zhang MQ Zhao K Combinatorial patterns of histone acetylations and methylations in the human genome Nat Genet 2008 11 897 903 18552846 10.1038/ng.154 PMC2769248

[B30] Creyghton MP Cheng AW Welstead GG Kooistra T Carey BW Steine EJ Hanna J Lodato MA Frampton GM Sharp PA Boyer LA Young RA Jaenisch R Histone H3K27ac separates active from poised enhancers and predicts developmental state Proc Natl Acad Sci U S A 2010 11 21931 21936 21106759 10.1073/pnas.1016071107 PMC3003124

[B31] Rada-Iglesias A Bajpai R Swigut T Brugmann SA Flynn RA Wysocka J A unique chromatin signature uncovers early developmental enhancers in humans Nature 2011 11 279 283 21160473 10.1038/nature09692 PMC4445674

[B32] Ishihama K Yamakawa M Semba S Takeda H Kawata S Kimura S Kimura W Expression of HDAC1 and CBP/p300 in human colorectal carcinomas J Clin Pathol 2007 11 1205 1210 17720775 10.1136/jcp.2005.029165 PMC2095491

[B33] Bustin SA Beaulieu J-F Huggett J Jaggi R Kibenge FS Olsvik PA Penning LC Toegel S MIQE précis: practical implementation of minimum standard guidelines for fluorescence-based quantitative real-time PCR experiments BMC Mol Biol 2010 11 74 20858237 10.1186/1471-2199-11-74 PMC2955025

[B34] Stypula-Cyrus Y Damania D Kunte DP Cruz MD Subramanian H Roy HK Backman V HDAC up-regulation in early colon field carcinogenesis is involved in cell tumorigenicity through regulation of chromatin structure PLoS ONE 2013 11 e64600 23724067 10.1371/journal.pone.0064600 PMC3665824

[B35] Huh JW Kim HC Kim SH Park YA Cho YB Yun SH Lee WY Chun H-K Prognostic impact of p300 expression in patients with colorectal cancer J Surg Oncol 2013 11 374 377 24142575 10.1002/jso.23405

[B36] Shechter D Dormann HL Allis CD Hake SB Extraction, purification and analysis of histones Nat Protoc 2007 11 1445 1457 17545981 10.1038/nprot.2007.202

[B37] Rizzardi AE Johnson AT Vogel RI Pambuccian SE Henriksen J Skubitz AP Metzger GJ Schmechel SC Quantitative comparison of immunohistochemical staining measured by digital image analysis versus pathologist visual scoring Diagn Pathol 2012 11 42 22515559 10.1186/1746-1596-7-42 PMC3379953

[B38] Mikula M Gaj P Dzwonek K Rubel T Karczmarski J Paziewska A Dzwonek A Bragoszewski P Dadlez M Ostrowski J Comprehensive analysis of the palindromic motif TCTCGCGAGA: a regulatory element of the HNRNPK promoter DNA Res 2010 11 245 260 20587588 10.1093/dnares/dsq016 PMC2920758

[B39] Käll L Storey JD MacCoss MJ Noble WS Assigning significance to peptides identified by tandem mass spectrometry using decoy databases J Proteome Res 2008 11 29 34 18067246 10.1021/pr700600n

[B40] Taus T Köcher T Pichler P Paschke C Schmidt A Henrich C Mechtler K Universal and confident phosphorylation site localization using phosphoRS J Proteome Res 2011 11 5354 5362 22073976 10.1021/pr200611n

[B41] Kasper B Lauridsen TB Optimizing the identification of citrullinated peptides by mass spectrometry: utilizing the inability of trypsin to cleave after citrullinated amino acids Journal of Proteomics & Bioinformatics 2013 11 288 295

[B42] Bakun M Karczmarski J Poznanski J Rubel T Rozga M Malinowska A Sands D Hennig E Oledzki J Ostrowski J Dadlez M An integrated LC-ESI-MS platform for quantitation of serum peptide ladders: application for colon carcinoma study Proteomics Clin Appl 2009 11 932 946 21136997 10.1002/prca.200800111

