# Supplementary material for: Histone H3 lysine 27 acetylation is altered in colon cancer
Source: Clin Proteomics. 2014 Jun 3;11(1):24. doi: 10.1186/1559-0275-11-24 (PMC4071346; doi:10.1186/1559-0275-11-24)
Supplement: Additional file 2: Figure S1 — An example MS/MS spectrum of the peptide K(Ac)SAPATGGVK derived from the H3 histone proteins family. The amino acid sequence of the peptide includes the lizyne K27 residue. The plot was generated using the ExpertSystemGui application available at (http://www.biochem.mpg.de/mann/tools/). Figure S2. Immunohistochemical staining of 10 matched normal and CRC tissue sections with use of antobody against H3K27Ac. Magnifications 100X and 400X. Figure S3. H3K27Ac mark level in quiescent and proliferating CRC cell lines. Cells cultured for 24 h with 10% or 0.5% FBS were harvested, histones isolated by acidic extraction and then 5 μg of protein was resolved by SDS-PAGE and electrotransferred to PVDF membrane. Blotted proteins were assessed by Western blot analysis using the antibodies to H3 (ab1791) or H3K27Ac (ab4729). Densitometric measurements were performed using OptiQuant image analysis software. H3K27Ac level was normalised to the signal from total H3 and presented of the chart. Figure S4. Analysis of mRNA expression levels of CBP, p300 and HDAC1 in individual tissue samples of 26 adenocarcinomas (CRC) and 24 healthy mucosa’s (NC). One microgram of total RNA was reverse-transcribed to generate cDNA and then qPCR was performed using SYBR Green I chemistry. Green horizontal bars indicate means and red whiskers indicate standard deviation. Differences were analyzed using the Mann–Whitney test. [file 1559-0275-11-24-S2.doc]

**Figure S1:** An example MS/MS spectrum of the peptide K(Ac)SAPATGGVK derived from the H3 histone proteins family. The amino acid sequence of the peptide includes the lizyne K27 residue. The plot was generated using the ExpertSystemGui application available at (http://www.biochem.mpg.de/mann/tools/).


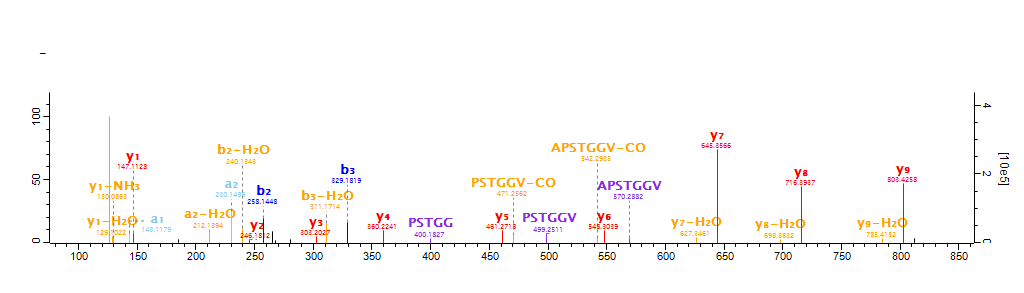


**Figure S2:** Immunohistochemical staining of 10 matched normal and CRC tissue sections with use of antobody against H3K27Ac. Magnifications 100X and 400X.


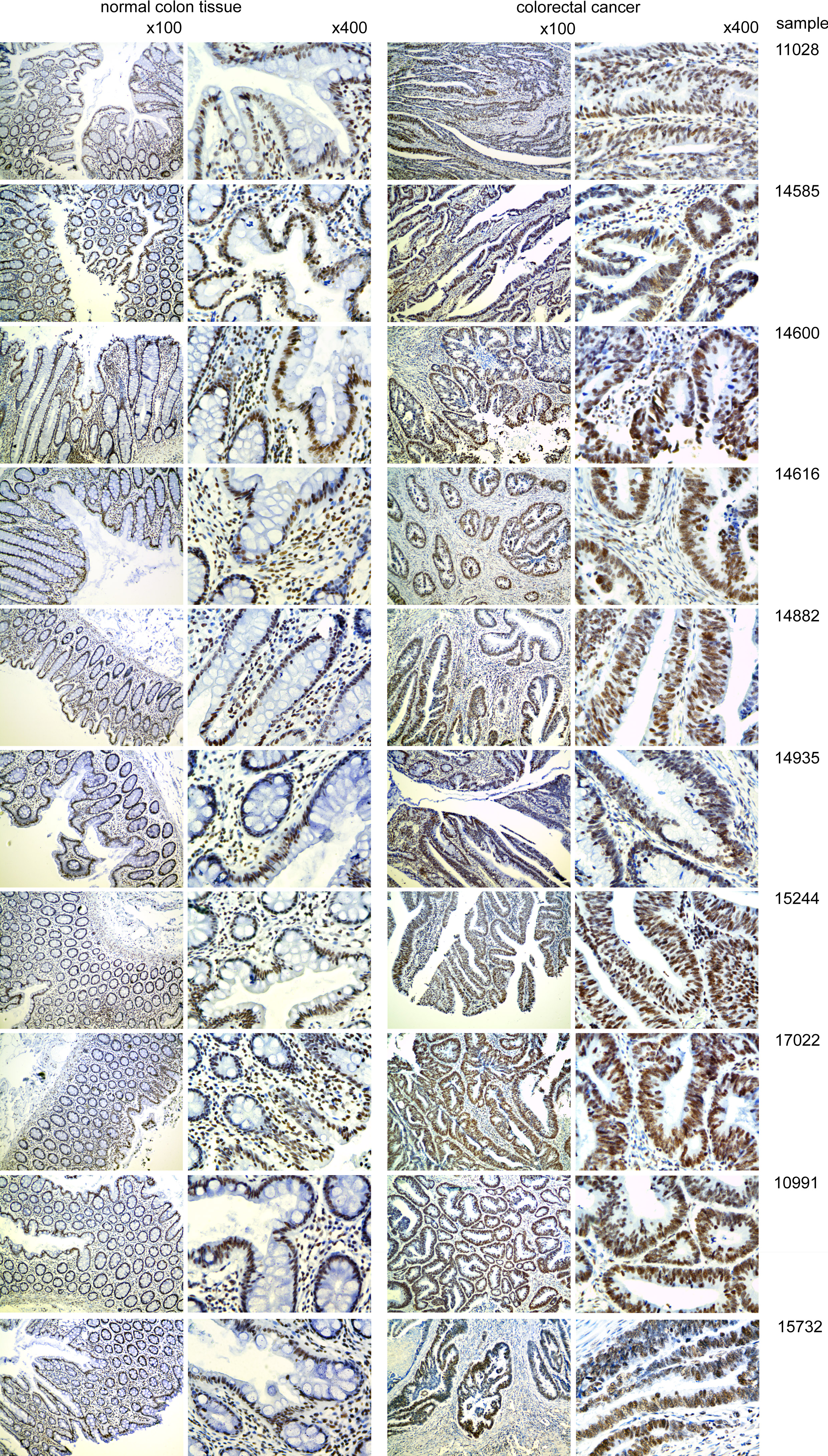


**Figure S3:** H3K27Ac mark level in quiescent and proliferating CRC cell lines. Cells cultured for 24h with 10% or 0.5% FBS were harvested, histones isolated by acidic extraction and then 5 µg of protein was resolved by SDS-PAGE and electrotransferred to PVDF membrane. Blotted proteins were assessed by Western blot analysis using the antibodies to H3 (ab1791) or H3K27Ac (ab4729). Densitometric measurements were performed using OptiQuant image analysis software. H3K27Ac level was normalised to the signal from total H3 and presented of the chart.

**Figure S4:** Analysis of mRNA expression levels of CBP, p300 and HDAC1 in individual tissue samples of 26 adenocarcinomas (CRC) and 24 healthy mucosa’s (NC). One microgram of total RNA was reverse-transcribed to generate cDNA and then qPCR was performed using SYBR Green I chemistry. Green horizontal bars indicate means and red whiskers indicate standard deviation. Differences were analyzed using the Mann-Whitney test.

**
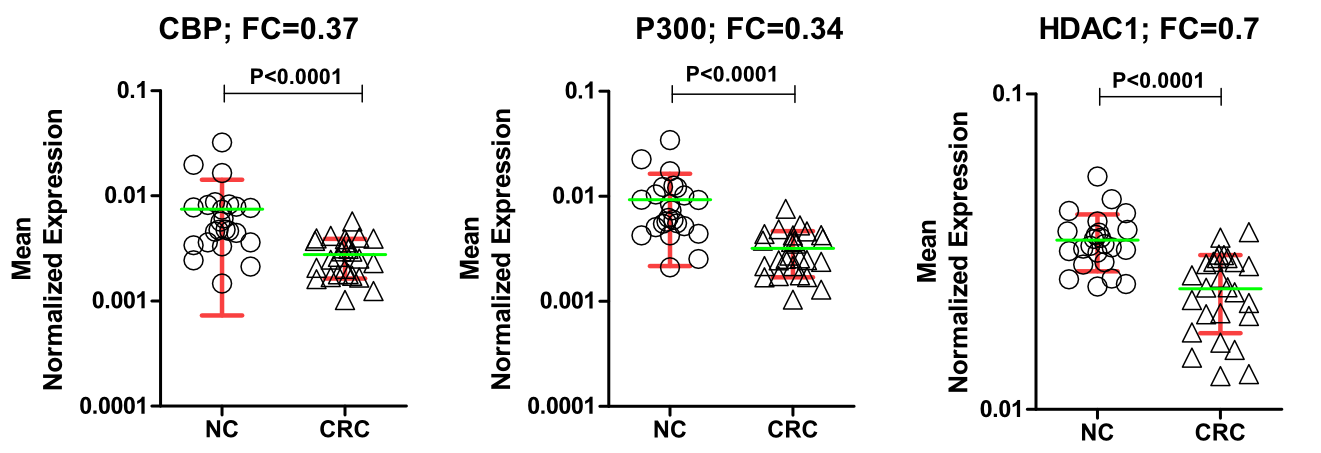
**
